# Pool testing on random and natural clusters of individuals: Optimisation of SARS-CoV-2 surveillance in the presence of low viral load samples

**DOI:** 10.1371/journal.pone.0251589

**Published:** 2021-05-18

**Authors:** Michela Baccini, Emilia Rocco, Irene Paganini, Alessandra Mattei, Cristina Sani, Giulia Vannucci, Simonetta Bisanzi, Elena Burroni, Marco Peluso, Armelle Munnia, Filippo Cellai, Giampaolo Pompeo, Laura Micio, Jessica Viti, Fabrizia Mealli, Francesca Maria Carozzi

**Affiliations:** 1 Department of Statistics, Computer Science, Applications, University of Florence, Florence, Italy; 2 Florence Center for Data Science, University of Florence, Florence, Italy; 3 Regional Laboratory of Cancer Prevention, Institute for Prevention, Research and Oncological Network (ISPRO), Florence, Italy; "INSERM", FRANCE

## Abstract

Facing the SARS-CoV-2 epidemic requires intensive testing on the population to early identify and isolate infected subjects. During the first emergency phase of the epidemic, RT-qPCR on nasopharyngeal (NP) swabs, which is the most reliable technique to detect ongoing infections, exhibited limitations due to availability of reagents and budget constraints. This stressed the need to develop screening procedures that require fewer resources and are suitable to be extended to larger portions of the population. RT-qPCR on pooled samples from individual NP swabs seems to be a promising technique to improve surveillance. We performed preliminary experimental analyses aimed to investigate the performance of pool testing on samples with low viral load and we evaluated through Monte Carlo (MC) simulations alternative screening protocols based on sample pooling, tailored to contexts characterized by different infection prevalence. We focused on the role of pool size and the opportunity to develop strategies that take advantage of natural clustering structures in the population, e.g. families, school classes, hospital rooms. Despite the use of a limited number of specimens, our results suggest that, while high viral load samples seem to be detectable even in a pool with 29 negative samples, positive specimens with low viral load may be masked by the negative samples, unless smaller pools are used. The results of MC simulations confirm that pool testing is useful in contexts where the infection prevalence is low. The gain of pool testing in saving resources can be very high, and can be optimized by selecting appropriate group sizes. Exploiting natural groups makes the definition of larger pools convenient and potentially overcomes the issue of low viral load samples by increasing the probability of identifying more than one positive in the same pool.

## Introduction

Since the first detection in Wuhan, China, in December 2019, severe acute respiratory syndrome coronavirus 2 (SARS-CoV-2), the pathogen of coronavirus disease 2019 (COVID-19), has spread worldwide to become a pandemic [1].

The most commonly used and reliable test to confirm the presence of SARS-CoV-2 is the Real Time-PCR performed on nasopharyngeal (NP) swabs or other respiratory tract specimens, amplifying viral RNA genes of the envelope (env), nucleocapsid (N), spike (S), RNA-dependent RNA polymerase (RdRp), and ORF1. The RT-qPCR on RNA extracted from NP swab is considered the gold standard to assess the presence of SARS-CoV-2 with a specificity of 100% and a sensitivity of 93–100% [2, 3]. However, several pre-analytical and analytical vulnerabilities may affect the stability of NP swabs reducing the test performances [4, 5]. For example, it is widely known that RNA is stable when stored at 2–8°C for up to 72 hours but data regarding the effect of storage at +4°C or at -20°C before or after virus inactivation on viral RNA detection has not been reported so far [5].

In China and Europe, during the epidemical emergency in the Spring 2020, acute shortage of reagents availability, as well as the correlated choice of performing RT-qPCR tests preferentially on symptomatic patients, led to significant underestimation of the actual infection burden, leaving many asymptomatic patients undetected [6, 7]. This has highlighted a heavy weakness in the system, stressing the need of developing screening procedures that require fewer resources and can be extended to larger portions of the population, thus allowing early detection and isolation of new cases.

RT-qPCR on samples obtained by pooling NP individual swabs seems to be a promising technique to improve surveillance. In fact, performing tests on pooled samples from a group of subjects and proceeding to single testing only on those groups resulted positive may save time and resources [8–14]. However, a crucial point in pool testing is that pooling may decrease the sensitivity of RT-PCR assays due to specimen dilution, leading to a higher rate of false negatives [15]. This issue appears to be particularly relevant in the presence of low viral load infections, as those observed during the second phase of the epidemic, when, after the acute phase of the emergency, positive cases at only one viral gene with high Cycle threshold (Ct is the number of replication cycles required to produce a detectable fluorescent signal; lower Ct values represents higher viral RNA loads) have been frequently observed, and in general when testing is extended to a large number of asymptomatic or pauci-symptomatic subjects. For this reason, it is a priority to understand if the sensitivity of the RT-PCR assays is preserved when pooling, and in particular to determine which is the maximum pool size that guarantees the expected sensitivity in a context of predominance of low viral load samples. Several studies have investigated RT-qPCR pool testing through laboratory experiments or in pilot studies [9, 12, 13, 15–20]. Some of them focused on the dilution effect and with few exceptions found that samples with Ct > 35 are not detectable even in pools of 5 samples [15, 18–20]. Bateman and colleagues [15], on the basis of laboratory analyses on pools with dilutions 1:5, 1:10, 1:50, concluded that Ct≥32 are sometimes not detectable even in pools of 5 and 10.

An apparently different but related issue concerns the setting up of tools to define appropriate screening protocols based on sample pooling, tailored to the specific real contexts in which they will be applied [10, 11, 14, 21–27]. In fact, the pool size which optimizes the gain with respect to single RT-PCR test could vary depending on the characteristics of the population on which pool testing is applied. The prevalence of infection and the presence of “natural” clusters of subjects (e.g. families, school classes, hospital rooms) on which the pool test could be performed are key features to determine the best testing strategy.

Our study wants to contribute to the discussion about the use of RT-qPCR pool testing through a reflection on its actual potentials in the second/third phase of the epidemic. In particular, our goal is twofold:

- providing new evidence from laboratory analyses about the performance of pool testing on samples with low viral load, which is still limited in the literature;
- exploring, through simulation algorithms, the performance of pool testing when applied on different hypothetical populations.

Regarding the first objective, we report the results of a preliminary analysis conducted on a small number of samples, aimed at investigating the COVID-19 RNA stability at different storage temperatures and times from collection, and the ability of the RT-qPCR pool testing to detect positive samples even with Ct above 35. Regarding the second objective, we performed Monte Carlo (MC) simulation analyses to evaluate the performance of pool testing when applied in pseudo-populations with different prevalence of infection. We focused on the role of pool size and on the opportunity to take advantage of the presence of a natural clustering structure in the population in order to increase the gain of the procedure in terms of saved RT-qPCR analyses.

## Methods

### Laboratory analyses

The laboratory analyses were performed on completely anonymous leftover samples from swabs of patients already analysed for the presence of SARS-CoV-2. Samples were collected by someone other than the authors (nursing personnel). No consent was obtained from patients, being the samples anonymized before we accessed them for the current analyses.

Each positive sample was retested singularly after anonymization and then included in the pools considering only the results from this second test for subsequent evaluations.

#### Sample collection

Nasopharyngeal swabs were collected using eSwab^®^ devices (Copan Italy) containing liquid Amies media. Samples were processed at the Regional Laboratory of Oncological Prevention Unit of the Institute for cancer prevention, research and oncological network (ISPRO), Florence (Italy).

#### Single sample test

Nasopharyngeal swabs were firstly tested as single samples, inactivating 300 μL of transport swab medium with 225 μL of lysis buffer and 15 μL of Proteinase K, incubated at 56°C for 15 minutes, immediately after arriving in lab.

Viral RNA was extracted using an automated system (NIMBUS IVD, Seegene) and the SARS-CoV-2 detection was performed by RT-real time PCR (Allplex^™^ 2019-nCoV Assay, Seegene; CFX96^™^, Bio-Rad) amplifying three viral genes (E, RdRP and N) and a process Internal Control (IC). The amplification occurred if Ct was inferior to 40 cycles. Results have been interpreted as follows: RNA virus was considered not detected if only IC was amplified; RNA virus was considered detected if at least one of the viral genes was amplified; RNA virus was considered detected with low viral load if one or more of the viral genes were amplified with a Ct>35.

#### SARS-CoV-2 RNA stability test

After performing the first RT-qPCR, the remaining swab transport media of 7 positive samples were processed in 3 different ways:

- Group A: remaining swab transport media were stored at −20°C and subsequently thawed and inactivated (4 samples: 1A, 2A, 3A, 4A).
- Group B: remaining swab transport media were inactivated after storing at 4°C for 8 hours and then frozen at -20°C (2 samples: 5B, 6B).
- Group C: remaining swab transport media was inactivated immediately when arrived in lab and then stored at -20°C (1 sample: 7C)

The 7 samples were re-tested after thawing, under the same analytical conditions of the first analysis.

#### Sample pooling

Firstly, we prepared 3 pools—one for each group of positive samples—mixing equal volumes of 10 negative nasopharyngeal swabs. Then 7 pools of 5 specimens and 7 pools of 10 specimens, each containing only one positive sample, were prepared. Specifically, for each positive sample (4 in A, 2 in B, 1 in C), we mixed 432 μL of negative pool and 108 μL of the positive sample to obtain a pool of 5 specimens and 486 μL of negative pool and 54 μL of the positive sample to obtain a pool of 10 specimens.

For samples 5B and 7C we tested also pools with 19 and 29 negative samples, diluting the pool of 10 samples 1:2 and 1:3 with the negative pool.

Each pool was then tested under the same conditions of the single sample.

### Monte Carlo simulations

Monte Carlo analyses were implemented considering a pseudo-population of N = 10000 subjects, on which a two-stage screening procedure is applied: first subjects are grouped and pool testing is performed on each group, then individual RT-qPCR tests are performed on individuals belonging to the groups that tested positive.

We considered 6×5 scenarios defined by the percentage of infected subjects in the population (prevalence, *p*) and the group size used for pool testing (*k*). Specifically, we considered *p* = 0.003, 0.005, 0.01, 0.03, 0.05, 0.1, and *k* = 2, 3, 4, 5, 10. The values of *p* were tailored to reproduce contexts of very low/moderate prevalence of ongoing infections.

For each scenario, we hypothesized two different strategies for group formation. With the first strategy (*R*), groups are randomly defined, i.e., they are composed by randomly partitioning the population into *n*_*g*_ *= N/k* groups. When the prevalence is small (i.e. the number of infected individuals is small compared to *n*_*g*_), this—with rare exceptions—leads to groups with at most a single infected individual.

With the second strategy (*C*), groups are generated assuming that they correspond to natural clusters in the population and, specifically that, if one member is infected, there is a higher likelihood that other group members are also infected. Examples of natural clusters in a real population are families, subjects sharing the same workplace, patients in the same room or in the same floor of a hospital, classmates. In order to generate these groups, we assumed that the number of positive subjects within each positive group of size *k* followed a zero-truncated Binomial distribution with parameters *k* and *π*, defined as follows:

- *k* is the number of experiments for the Binomial distribution,
- *π* is the probability of success for the Binomial distribution, which represents the probability of having ongoing infection for a subject belonging to a cluster where there is at least one infected subject.

For each *k*, we considered different values of *π*: the larger the value of *π*, the higher the likelihood that infected subjects tend to concentrate in the same groups is. As a consequence, as *π* increases, the number of positive groups decreases. It is worth noting that each combination of *k* and *π* produces a different expected number of positive subjects within each positive group, corresponding to the expected value of the zero-truncated Binomial distribution, *πk*/(1 − (1 − *π*)*k*). Specifically, for each *k*, we considered values of *π* such that the expected number of positive subjects within each positive group ranged between low (less than 2) and quite high values (90% of *k*). Even if for each scenario, i.e. each pair of *k* and *π*, the expected number of infected specimens was equal for all positive groups, the observed number could vary from 1 to *k*.

From a practical point of view, simulations were performed iteratively by randomly sampling from a Binomial distribution of parameters *k* and *π* the number of infected individuals to be assigned to the first group of *k* individuals, then to the second one and so on, until there were no more infected individuals to be assigned to a pool. All the remaining groups were assumed to include zero infected specimens. Since positive groups contain at least one infected individual, the actual distribution of the number of infected individuals in these groups is a zero-truncated Binomial distribution.

Taking the individual RT-qPCR test as the gold standard, in our simulation we assumed that the specificity of pool testing was nearly optimal and equal to the one estimated by Hogan and colleagues in one of the first paper that focused on pool testing [9]: they found only 1 positive over 290 pools of uninfected samples, for a specificity of the results on the pool (probability that a pool is negative given that it does not include positive specimens) equal to 0.997. Regarding sensitivity (probability that a pool is positive given that it includes at least one positive specimen), we focused on an optimal situation where sensitivity of pool testing was very high regardless of the pool size, and in particular equal to 0.995 [16]. This sensitivity value is likely appropriate in populations where the viral loads of the infected subjects are high. For example, in Bateman et al. [15] viral loads with Ct≤28 were always detected in both 1:5 and 1:10 dilutions.

In order to get some insights on the role of the dilution effects, we also performed simulations under the assumption that the sensitivity of pool testing decreases as the pool size increases when groups are randomly constructed (see S1 Appendix for details and results).

For each *p* and *k* and each pool testing procedure, we ran 500 MC iterations and for each iteration, we calculated the following quantities: number of RT-qPCR tests (total number of pool tests performed at the first step plus individual tests performed at the second step on the positive groups), percentage of saved RT-qPCR tests, defined as 100×(1-number of RT-qPCR tests/N), number of individuals receiving a false negative result and probability that a subject receiving a negative result is actually not infected (negative predictive value, NPV). Note that, under the assumption that the individual RT-qPCR test is the gold standard, the pool testing procedure does not lead to false positive results—individuals belonging to false positive groups are correctly classified at the second step of the procedure when individual tests are performed—and the positive predictive value is 1.

For each simulation setting, we calculated the MC mean and 90% variability interval (5^th^ and 95^th^ percentiles of the simulated values) for each quantity of interest.

## Results

### Laboratory analyses

After thawing, the samples from group A showed a reduction of viral load compared to the first test, with an increase in Ct ranging from 0.67 to 4.53. The ΔCt (Ct at the re-test—Ct at the first test) was greater as the viral load was lower. The viral load decreased also in the 5B sample, but the increase in Ct was less evident. Samples 6B and 7C maintained the same viral load in the repeated test (Table 1).

**Table 1:** Viral load measured in terms of Ct for the three amplified genes (E, RdRp, N) from RT-qPCR analyses on single specimens and on pools of 5, 10, 20 and 30 samples with only one positive.

	Sample		Ct from RT-qPCR			ΔCt		
			gene E	gene RdRp	gene N	gene E	gene RdRp	gene N
**Group A**	1A	First test	N/A	34.41	35.80	-	3.21	3.94
		Re-test	N/A	37.62	39.74			
		pool of 5 samples	N/A	N/A	N/A			
		pool of 10 samples	N/A	N/A	N/A			
	2A	First test	25.41	26.69	26.92	1.34	1.33	1.28
		Re-test	26.75	28.02	28.20			
		pool of 5 samples	28.03	29.85	29.73			
		pool of 10 samples	29.42	31.04	31.4			
	3A	First test	16.66	17.21	19.33	1.41	2.11	0.67
		Re-test	18.07	19.32	20.00			
		pool of 5 samples	20.52	22.12	22.09			
		pool of 10 samples	22.18	23.12	23.04			
	4A	First test	22.54	24.20	24.98	3.9	3.23	4.53
		Re-test	26.44	27.43	29.51			
		pool of 5 samples	27.28	28.58	28.79			
		pool of 10 samples	27.66	29.18	29.34			
**Group B**	5B	First test	29.83	31.36	31.48	0.77	0.7	-0.49
		Re-test	30.60	32.06	30.99			
		pool of 5 samples	N/A	35.23	32.99			
		pool of 10 samples	N/A	36.68	34.60			
		pool of 20 samples	N/A	38.09	36.66			
		pool of 30 samples	N/A	N/A	38.08			
	6B	First test	N/A	36.70	35.97	-	0.59	-0.77
		Re-test	N/A	37.29	35.20			
		pool of 5 samples	N/A	37.41	38.05			
		pool of 10 samples	N/A	N/A	N/A			
**Group C**	7C	First test	23.60	26.35	27.03	0.04	-1.33	-0.54
		Re-test	23.64	25.02	26.49			
		pool of 5 samples	26.42	28.27	27.94			
		pool of 10 samples	26.59	27.63	28.16			
		pool of 20 samples	29.95	31.73	34.04			
		pool of 30 samples	30.34	32.12	34.12			

N/A: not amplified

ΔCt: difference in Ct after and before thawing.

Samples with high viral load (Ct<30 for at least one viral gene) were detectable even in pool with 29 negative samples, if nasopharyngeal swabs were inactivated before freezing (group C).

Viral RNA was identifiable in pool with 29 negative swabs even in sample 5B, which showed an intermediate viral load (Ct between 30 and 35); however, the virus identification seemed to be less reliable, considering that only one gene remained detectable with a very high Ct (38.08).

Sample 6B, with a really low viral load (Ct>35 for all genes), was detectable only in pool with 4 negative samples. Unfortunately, the scarce material available for this sample did not allow to analyse intermediate pool dimensions.

### Monte Carlo simulations

As expected, under the pool testing strategy *R* the average number of positive groups and the average number of infected subjects per group increased with increasing *p* and/or *k* (Table 2).

**Table 2:** Pool testing on random groups: Monte Carlo (MC) mean of the number of positive subjects within each positive group and of the number of positive groups, by prevalence (*p*) and group size (*k*).

*k*	Number of	MC mean					
		*p = 0*.*003*	*p = 0*.*005*	*p = 0*.*01*	*p = 0*.*03*	*p = 0*.*05*	*p = 0*.*1*
*2*	Positive subjects per positive group	1.00	1.00	1.01	1.02	1.03	1.05
	Positive groups	30	50	100	296	488	949
*3*	Positive subjects per positive group	1.00	1.01	1.01	1.03	1.05	1.11
	Positive groups	30	50	99	291	475	903
*4*	Positive subjects per positive group	1.01	1.01	1.02	1.05	1.08	1.16
	Positive groups	30	50	99	287	464	860
*5*	Positive subjects per positive group	1.01	1.01	1.02	1.06	1.10	1.22
	Positive groups	30	50	98	283	453	820
*10*	Positive subjects per positive group	1.01	1.02	1.05	1.14	1.25	1.54
	Positive groups	30	49	96	263	402	651

Population size = 10000.

Table 3 summarizes the group composition under the pool testing strategy *C*. In particular, for each value of *k* and *π*, it reports the expected number of positive subjects within each positive group and, for each prevalence *p*, the corresponding MC mean of the number of positive groups. Of course, given the expected number of positive subjects within each positive group, the number of positive groups increased with increasing prevalence.

**Table 3:** Pool testing on natural clusters: Expected number of positive subjects within each positive group, by group size (*k*) and Binomial probability (*π*), and Monte Carlo mean of the number of positive group by group size (*k*), Binomial probability (*π*) and prevalence (*p*).

*k*	*π*	Expected number of positive subjects per positive group	MC mean of the number of positive groups					
			*p = 0*.*003*	*p = 0*.*005*	*p = 0*.*01*	*p = 0*.*03*	*p = 0*.*05*	*p = 0*.*1*
2	0.1	1.05	28.6	47.5	95.1	285.3	475.1	938.6
	0.2	1.11	27.0	45.2	90.2	270.2	450.3	899.6
	0.3	1.18	25.5	42.4	85.1	255.4	424.7	849.8
	0.4	1.25	24.2	39.8	80.4	239.9	399.7	800.1
	0.5	1.33	22.7	37.8	75.3	225.3	375.0	750.5
	0.6	1.43	21.1	35.3	70.3	209.9	350.1	700.1
	0.7	1.54	19.8	32.7	65.2	195.1	325.1	649.9
	0.8	1.67	18.2	30.2	60.2	180.7	300.4	600.1
	0.9	1.82	16.7	27.8	55.2	165.4	275.1	550.3
3	0.1	1.11	27.1	45.1	90.5	270.8	451.8	892.8
	0.2	1.23	24.6	40.9	81.7	244.3	406.7	813.5
	0.3	1.37	22.0	36.8	73.2	219.7	365.7	731.2
	0.4	1.53	19.8	33.1	65.5	196.4	327.0	653.0
	0.5	1.71	17.8	29.5	58.7	175.4	291.7	584.4
	0.6	1.92	15.8	26.3	52.4	156.3	260.6	520.4
	0.7	2.16	14.2	23.6	46.6	139.2	232.2	463.4
	0.8	2.42	12.7	21.1	41.6	124.4	206.9	413.7
	0.9	2.70	11.4	18.9	37.3	111.2	185.2	370.4
4	0.1	1.16	25.9	43.0	86.2	258.5	430.2	849.9
	0.2	1.36	22.3	37.2	73.9	221.6	369.2	738.0
	0.3	1.58	19.2	32.1	63.8	190.1	317.4	633.4
	0.4	1.84	16.5	27.4	54.8	163.6	272.7	544.7
	0.5	2.13	14.4	23.7	47.2	140.9	235.3	469.0
	0.6	2.46	12.5	20.6	40.8	121.9	203.3	406.4
	0.7	2.82	11.0	18.1	35.9	106.9	177.2	354.7
	0.8	3.21	9.8	16.0	31.5	93.8	156.3	312.4
	0.9	2.60	8.8	14.3	28.1	83.8	139.1	278.0
5	0.1	1.22	24.8	41.2	82.0	246.0	409.7	810.4
	0.2	1.49	20.5	33.9	67.5	202.2	336.7	672.7
	0.3	1.80	16.9	28.1	55.9	166.5	277.6	554.8
	0.4	2.17	14.2	23.4	46.4	139.0	231.0	461.7
	0.5	2.58	12.0	19.7	39.2	116.2	194.3	388.1
	0.6	3.03	10.3	16.9	33.4	99.4	165.6	330.7
	0.7	3.51	9.0	14.6	29.0	86.0	142.9	285.1
	0.8	4.00	7.9	12.9	25.4	75.5	125.4	250.6
	0.9	4.50	7.1	11.5	22.7	67.1	111.5	222.6
10	0.1	1.54	19.7	32.8	65.2	196.1	325.9	644.4
	0.2	2.24	13.9	22.7	45.0	134.4	223.9	446.9
	0.3	3.09	10.0	16.6	32.8	97.5	162.5	324.4
	0.4	4.02	7.9	13.0	25.3	75.0	124.2	249.3
	0.5	5.00	6.4	10.5	20.5	60.4	100.3	200.1
	0.6	6.00	5.5	8.8	17.2	50.4	83.8	166.9
	0.7	7.00	4.7	7.6	14.7	43.3	71.9	143.3
	0.8	8.00	4.1	6.7	12.9	38.0	62.9	125.4
	0.9	9.00	4.0	6.0	11.5	33.8	56.0	111.6

Population size = 10000.

Table 4 shows, for the random pool testing strategy, MC means and 90% variability intervals of the number of RT-qPCR tests, by prevalence and group size, assuming a sensitivity of pool testing equal to 0.995 regardless of *k* (no dilution effects). In populations where the proportion of infected individuals was lower than 1%, the total number of RT-qPCR tests progressively reduced as the group size increased, with rather tight non-overlapping 90% variability intervals. Moreover, the lower the prevalence of the disease, the lower the total number of RT-qPCR tests was. For populations with prevalence equal to 3%, our simulations still suggest an inverse relationship between *k* and the total number of required RT-qPCR tests as long as *k*<5, while for group sizes of 5 and 10 subjects the expected number of tests was similar. In populations where the prevalence was 5% or 10% the best group size ranged between 3 and 5; *k* = 10 was not recommendable, as it implied the highest number of RT-qPCR tests.

**Table 4:** Pool testing on random groups: Monte Carlo means^a^ (90% variability intervals) of the number of RT-qPCR tests, by prevalence (*p*) and group size (*k*).

*k*	MC mean of the number of tests					
	*p = 0*.*003*	*p = 0*.*005*	*p = 0*.*01*	*p = 0*.*03*	*p = 0*.*05*	*p = 0*.*1*
*2*	5089	5128	5227	5616	5997	6913
	(5076; 5100)	(5116; 5140)	(5214; 5238)	(5602; 5630)	(5980; 6014)	(6888; 6938)
*3*	3452	3511	3658	4230	4779	6052
	(3436; 3469)	(3496; 3529)	(3643; 3676)	(4207; 4252)	(4750; 4804)	(6010; 6094)
*4*	2648	2726	2920	3668	4370	**5941**
	(2628; 2668)	(2708; 2744)	(2900; 2940)	(3636; 3700)	(4332; 4408)	**(5880; 6000)**
*5*	2177	2274	2515	**3431**	**4276**	6091
	(2160; 2200)	(2255; 2295)	(2490; 2540)	**(3395; 3470)**	**(4225; 4325)**	(6010; 6170)
*10*	**1322**	**1513**	**1977**	3635	5013	7490
	**(1300; 1360)**	**(1480; 1550)**	**(1930; 2020)**	(3540; 3730)	(4890; 5130)	(7330;7640)

Population size = 10000; Specificity of pool testing = 0.997; Sensitivity of pool testing = 0.995.

(a) In bold the strategy which minimizes the number of tests, given the prevalence.

All the pool testing strategies in Table 4 appeared to perform well in terms of cases detection. Taking the individual RT-qPCR test as the gold standard, the NPVs were greater than 0.999 for values of prevalence and group sizes within the ranges considered in this analysis (results not reported). The expected number of false negatives (which represents a constant proportion of the total number of cases in each population) obviously increased with the prevalence: in the simulated population of 10000 it varied between 0.2 for *p* = 0.003 to 5 for *p* = 0.1 (see S1 Table).

**Fig 1:**
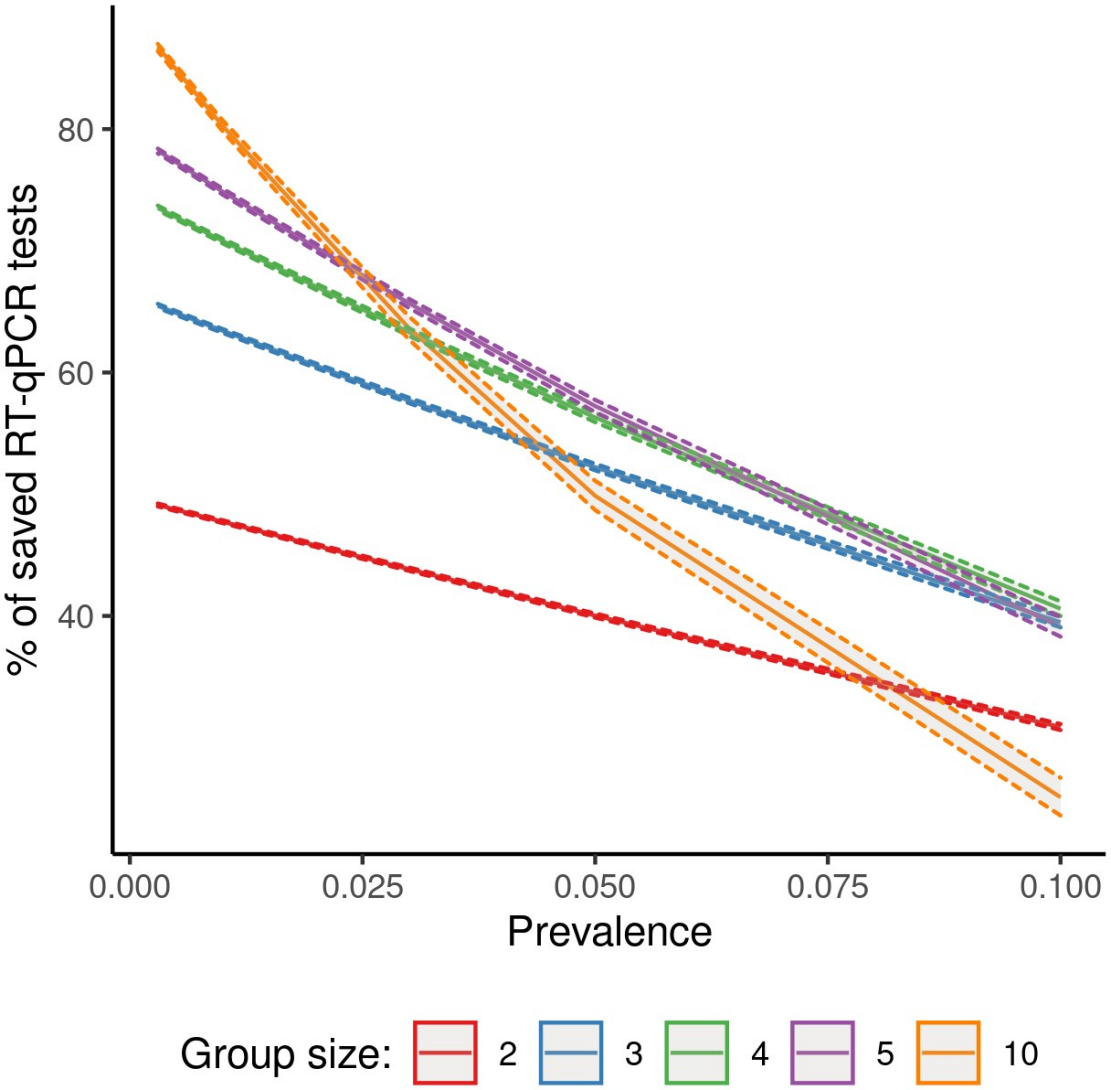
Pool testing on random groups: Monte Carlo means (solid lines) and 90% variability intervals (dashed lines) of the percentage of saved RT-qPCR tests by prevalence (*p*) and group size (*k*). Population size = 10000; Specificity of pool testing = 0.997; Sensitivity of pool testing = 0.995.

Fig 1 shows the MC means and 90% variability intervals of the percentage of saved RT-qPCR tests in respect to the gold standard procedure for the random strategy *R*. In populations where the proportion of infected individuals was lower than 1%, the pool-testing strategies reduced the number of required RT-qPCR test by more than 47% and the larger the group size, the higher the percentage of saved RT-qPCR tests was. Specifically, the main gain was obtained by applying the pool-testing with groups of size 5 and 10, which led to save more than 74% and 80% of RT-qPCR tests, respectively. In populations where the proportion of infected individuals was greater than (or equal to) 3%, our results suggest that the best pool-testing strategies were those with *k* ranging between 4 and 5.

**Fig 2:**
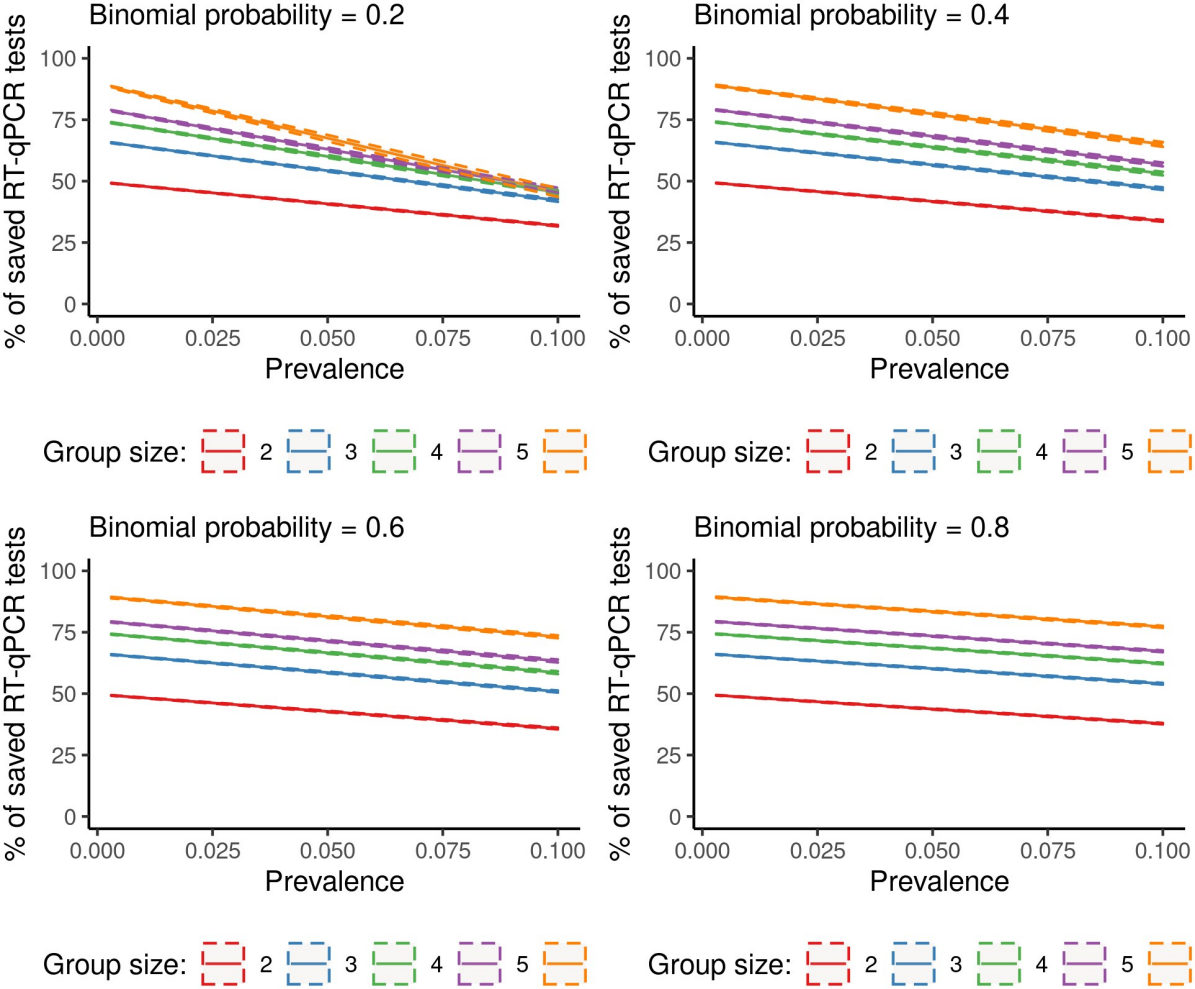
Pool testing on natural clusters: Monte Carlo means (solid lines) and 90% variability intervals (dashed lines) of the percentage of saved RT-qPCR tests by group size (*k*), Binomial probability (*π*) and prevalence (*p*). Population size = 10000; Specificity of pool testing = 0.997; Sensitivity of pool testing = 0.995.

Each panel in Fig 2 shows the percentage of saved RT-qPCR tests as a function of prevalence, *p*, and group size, *k*, in the case of natural clusters. The four panels refer to four scenarios of *π*, which tunes the natural correlation between individuals in the same group. Taking advantage of this natural correlation, pools of 10 subjects provided a gain even in situations where the prevalence was relatively high (5%).

## Discussion

The pandemic of SARS-CoV-2 represents a hard challenge for health-care systems and their facilities. Expanded molecular testing for SARS-CoV-2 is urgently needed to enable identification of infected individuals, tracing and quarantining of their contacts. Since the beginning of the emergency, the strategy of pool testing has been proposed as an alternative to RT-qPCR single tests to save time and resources [8, 9, 12, 13]. However, sample pooling determines a dilution of primary specimens, which may cause the non-detection of samples with low viral load, which could represent a large number of cases in some phases of the epidemic. Moreover, there is not only one way to perform pool testing, as different strategies for groups creation can be used, that can be more or less complex and more or less convenient depending on the actual epidemic scenario [14].

In this study, we firstly tested the stability of nasopharyngeal swabs and the ability of detecting positive samples with low viral load in pools of different size. Then, we investigated through simulations the performance of alternative pool testing strategies, quantifying the potential gain in terms of saved RT-pPCR tests as the pool size changed, under different prevalence scenarios. Specifically, we focused on strategies where the groups were built at random and strategies that exploited the possible presence of natural clusters of infection in the population. While studies have been published which investigate the first kind of scenarios [21–27], the possible advantages of performing pool testing on natural clusters have been less explored. In a recent paper, Reweley and colleagues [11] come to conclusions similar to ours. They do not address either the problem of dilution or false negative results, but show via simulations that pool testing could be more efficient than expected even for high levels of prevalence, provided that groups are constructed following the order of the specimens collected in the same sampling site. This order could in fact reflect the presence of infection clusters in the population.

The results of the laboratory analyses should be interpreted with caution because conducted on a limited number of specimens. However, they suggest that, while high viral load samples seem to be detectable even in pool with 29 negative samples, particular attention should be deserved to specimens with high Ct: we were able to identify one sample with a Ct of 37.3 only in pool with 4 negative specimens. This is in line with results reported elsewhere which show a decreased sensitivity of pool testing in the presence of low viral load samples [15, 18, 19].

The experimental results also indicate that inactivating nasopharyngeal eSwab^®^ devices as soon as possible and, in any case, before freezing the samples, is fundamental. We did not investigate the performance of RT-qPCR of samples stored at -80°C before inactivation, as suggested by CDC guidelines [28]. However, Torres and colleagues [18], using samples stored at -80°C before inactivation, were not able to detect samples with Ct>35 even in mini-pool of 5 samples. So we can speculate that inactivating the nasopharyngeal swabs before freezing remains fundamental even in case of storing at -80°C.

The MC simulations indicate that the accommodation of the pool size to different infection scenarios is challenging and should be carefully considered when planning screening strategies based on pool testing. Overall, our results confirm that pool testing is useful in epidemic phases or contexts where the infection prevalence is low. In these situations, the gain of using pool testing in respect to individual RT-qPCR tests can be very high in terms of saved resources, and can be optimized selecting an appropriate group size. If a random criterion is used for group creation, pool testing on groups of 10 specimens could be in principle very convenient for values of prevalence up to 0.01, but smaller pools of 4 or 5 specimens should be preferred as the prevalence increases. On the contrary, if pool testing is conducted on natural clusters of infection, pools of 10 specimens could still be a good choice even for prevalence exceeding 0.01.

In our main MC simulations, we assumed very high sensitivity for pool testing, obtaining a very low number of false negatives even in case of large prevalence. However, as already discussed, both our experimental results and evidences reported in the literature indicate that the sensitivity could be not optimal in case of low viral load samples, especially for large *k* [15, 18–20]. Nevertheless, it is worth noting that defining the sensitivity of pool testing as a function of pool size is a highly speculative exercise. Even if laboratory results were available to derive the probability of detecting viral loads in pools of different size as a function of Ct—this is the idea in Bateman et al. [15]–, or if mathematical models for the dilution effect had been developed [29], realistic simulations would require hypotheses about the distribution of the viral loads, which may change across populations and over time.

While a reduction of the sensitivity of pool testing as *k* increases is expected if the groups are randomly created and, consequently, the expected number of positive specimens in the positive pools is around 1 (for values of ongoing infections prevalence within the range considered in this paper), this could be not the case if pool testing is performed on natural groups (e.g. families). In fact, if pool testing is performed on natural groups, the probability of more than one positive specimen in the same group is higher and this likely leads to an increase in the expected overall viral load of the pools containing infected individuals, making the virus detectable even if the viral loads of the single swabs are low. This could be an additional advantage of applying pool testing strategies which exploit natural clusters in the population or, more in general, which tend to put together subjects with larger probability of being infected.

In balancing the gain in terms of saved time and resources with the accuracy of pool testing, it is crucial to assess the actual public health consequences of leaving low viral load infections undetected. In fact, a positive PCR result reflects the presence of viral RNA but does not necessarily indicate the identification of viable virus. Although viral RNA can be detected by PCR even after the resolution of symptoms, the amount of detected viral RNA is substantially reduced over time and generally below the threshold where replication competent virus can be isolated [30–33]. Wölfel and colleagues [30] found that virus isolation is not successful beyond the 8^th^ day from illness onset, when the viral load dramatically decreases, and <6 log10 RNA was previously shown to represent the viral RNA load threshold for virus infectivity.

Additionally, some studies documented that there may be a correlation between reduced infectivity and decrease in viral loads or did not rule out it [34, 35]. This hypothesis has led the WHO to review the “Criteria for releasing COVID-19 patients from isolation” on June 17^th^ 2020 [36]. If, according to these evidences, larger Ct are indicative of lower infectivity, the reduced sensitivity of pool testing with respect to individual testing in detecting low viral loads would have less severe consequences.

Finally, we would like to remark that creating pools according to natural clusters in the population could provide an additional guarantee that any false negative groups have a negligible impact in terms of public health. In fact, infectivity of a subject with low viral load belonging to a natural group (e.g. family), where all other members are negative, is likely under the threshold allowing the contagion. Thus, if the low viral load is not detected because of the unfavourable specimen dilution (only one infected over *k*), the risk that new infections may derive from the undetected one could be low. On the contrary, if a low viral load is able to produce contagion, more than one infected specimen is expected in the natural cluster, with the consequence of a larger probability that the pool test results positive.

## Conclusions

RT-qPCR pool testing can be a cost-effective procedure to perform screening on populations where the prevalence of ongoing infection is low.

Dilution of the specimens is a crucial issue and further investigation is needed to define testing procedures which minimize the degradation of viral RNA before sample pooling.

Exploiting the natural clusters in the population (e.g. families, school classes, hospital rooms) may enhance pool testing performance also in the presence of high rates of low viral load infections, allowing the definition of larger pools and increasing the gain in time and resources with respect to single RT-qPCR testing.

Assessing and comparing the performance of alternative screening procedures by simulations is a fundamental step before any practical implementation on real populations.

## Supporting information

S1 Appendix Simulations under a hypothetical scenario of dilution effect. (PDF)

S1 Table Pool testing on random groups: Monte Carlo means (90% variability intervals) of the number of false negatives, by prevalence (*p*) and group size (*k*). Population size = 10000; Specificity of pool testing = 0.997; Sensitivity of pool testing = 0.995.
(PDF)
