# Supplementary material for: Pool testing on random and natural clusters of individuals: Optimisation of SARS-CoV-2 surveillance in the presence of low viral load samples
Source: PLoS One. 2021 May 18;16(5):e0251589. doi: 10.1371/journal.pone.0251589 (PMC8130965; doi:10.1371/journal.pone.0251589)
Supplement: S1 Appendix — (PDF) [file pone.0251589.s001.pdf]

S1 Appendix. Simulations under a hypothetical scenario of dilution effect

In order to get some insights on the role of the dilution effects, we also performed simulations under the assumption that the sensitivity of pool testing decreases as the pool size increases. In particular, focusing on pools of 5 and 10 specimens, we assumed that the sensitivity of pool testing in the case of random groups was 0.93 and 0.91 for k=5 and k=10, respectively. These values are those estimated in Bateman et al. [1] for a real population of SARS-CoV-2 positive specimens with different levels of Ct, collected at the Wisconsin State Laboratory of Hygiene during the first epidemic wave. It is important to stress that because these values of sensitivity were derived for a population characterized by a specific viral loads distribution, they should not be considered as universally valid.

In case of natural clusters, we did not assume a decrease in sensitivity as the pool size increases, because when k=5 and k=10 the expected number of infections within positive groups resulted to be larger than one (Table 2 in the main text), a condition that in a real context would likely bring to higher viral loads at the group level.

Assuming a sensitivity of pool testing equal to 0.93 for k=5 and 0.91 for k=10 when grouping is random, the expected number of RT-qPCR tests required by the procedure slightly decreased when compared with the corresponding values reported in Table 4 in the main text, due to the presence of more false negative results. Therefore, in order to make a fair comparison between procedures having different sensitivity levels, the expected numbers of required RT-qPCR tests and the NPVs should be jointly considered. As an example, in Figure S1 we compared for k=2, 5, 10:

- pool testing on random groups in hypothetical populations where the infected individuals have high viral loads (no dilution effect, sensitivity=0.995 regardless of k),
- pool testing on random groups in hypothetical populations similar to the one investigated in Bateman et al. [1], characterized by a mixture of low and high viral loads (sensitivity=0.93 and 0.91 for k=5 and 10, respectively)
- pool testing on natural clusters in populations with any viral load distribution (no dilution effects, sensitivity=0.995, π=0.4).

Each panel in Figure S1 refers to a hypothetical population defined by p=0.005, 0.01, 0.05. In populations with low viral loads, the dilution effect may strongly reduce the cost-effectiveness of pool testing on random groups, reducing both the percentage of saved RT-qPCR tests and the negative predictive values, especially when p=0.01. These results further stress the benefits of using pool testing on natural clusters, which we can reasonably assume not to suffer from the problem of a reduced sensitivity.

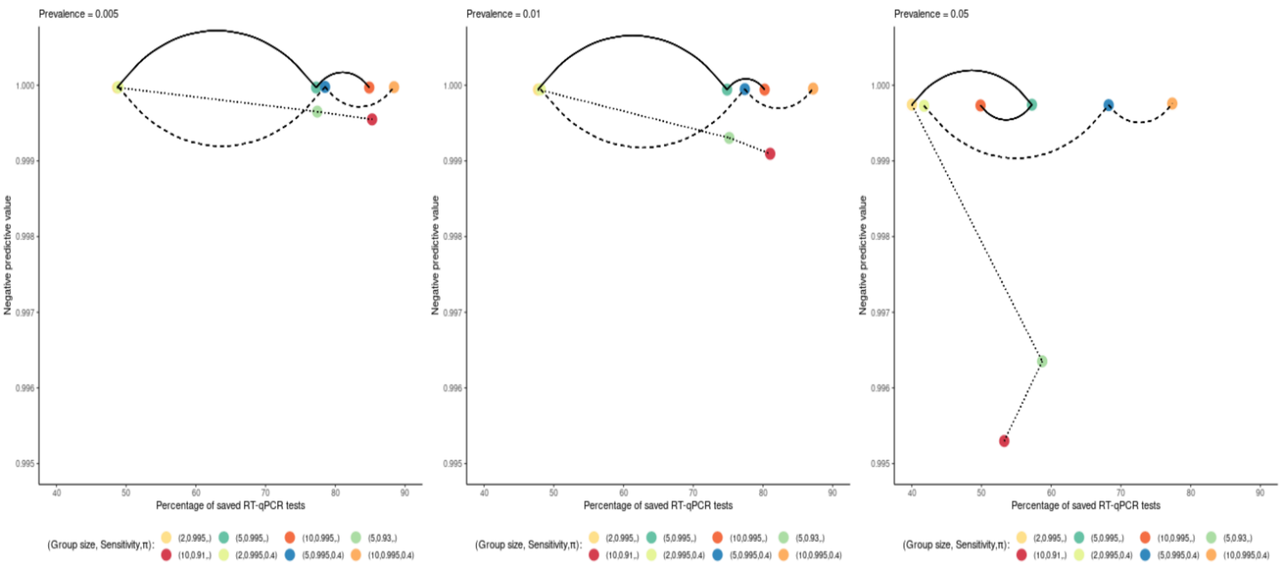

Figure S1. Monte Carlo mean of the percentage of saved RT-qPCR tests and the Negative Predictive Value when using alternative pool testing procedures on different hypothetical populations, by infection prevalence (*p*=0.005, 0.01, 0.05). Solid lines: pool testing on random groups in hypothetical populations where infected individuals have high viral loads (sensitivity=0.995 regardless of the group size *k*); Dotted lines: pool testing on random groups in hypothetical populations characterized by a mixture of low and high viral loads (sensitivity=0.93 for group size *k*=5; sensitivity=0.91 for group size *k*=10); Dashed lines: pool testing on natural clusters on any kind of population (sensitivity=0.995 regardless of the group size *k*).

References

1. Bateman AC, Mueller S, Guenther K, Shult P. Assessing the dilution effect of specimen pooling on the sensitivity of SARS-CoV-2 PCR tests. J Med Virol. 2020;1-5. https://doi.org/10.1002/jmv.26519
