# Supplementary material for: Pool testing on random and natural clusters of individuals: Optimisation of SARS-CoV-2 surveillance in the presence of low viral load samples
Source: PLoS One. 2021 May 18;16(5):e0251589. doi: 10.1371/journal.pone.0251589 (PMC8130965; doi:10.1371/journal.pone.0251589)
Supplement: S1 Table — Population size = 10000; Specificity of pool testing = 0.997; Sensitivity of pool testing = 0.995. (PDF) [file pone.0251589.s002.pdf]

S1 Table. Pool testing on random groups: Monte Carlo means (90% variability intervals) of the number of false negatives, by prevalence (p) and group size (k). Population size = 10000; Specificity of pool testing = 0.997; Sensitivity of pool testing = 0.995.

k	MC mean of the number of false negatives					
	p=0.003	*p=0.005*	*p=0.01*	*p=0.03*	*p=0.05*	*p=0.1*
2	0.2<br>(0.0; 1.0)	0.3<br>(0.0; 1.0)	0.6<br>(0.0; 2.0)	1.6<br>(0.0; 4.0)	2.4<br>(0.0; 5.0)	4.9<br>(2.0; 0.9)
3	0.2<br>(0.0; 1.0)	0.3<br>(0.0; 1.0)	0.6<br>(0.0; 2.0)	1.5<br>(0.0; 4.0)	2.4<br>(0.0; 5.0)	4.9<br>(1.0; 0.9)
4	0.2<br>(0.0; 1.0)	0.3<br>(0.0; 1.0)	0.6<br>(0.0; 2.0)	1.6<br>(0.0; 4.0)	2.5<br>(0.0; 5.0)	4.9<br>(2.0; 0.9)
5	0.2<br>(0.0; 1.0)	0.3<br>(0.0; 1.0)	0.6<br>(0.0; 2.0)	1.6<br>(0.0; 4.0)	2.5<br>(0.0; 5.0)	5.0<br>(2.0; 0.9)
10	0.2<br>(0.0; 1.0)	0.3<br>(0.0; 1.0)	0.6<br>(0.0; 2.0)	1.5<br>(0.0; 4.0)	2.5<br>(0.0; 6.0)	5.0<br>(1.0; 10.0)
